# Digital Long Covid Communities—Shaping Diagnoses, Treatment Strategies and Modes of Expertise

**DOI:** 10.1111/hex.70279

**Published:** 2025-04-28

**Authors:** Petter Almqvist‐Ingersoll

**Affiliations:** ^1^ Department of Thematic Studies Linköping University Linköping Sweden

**Keywords:** epistemology, long Covid, netnography, social media, sociology of diagnosis

## Abstract

**Introduction:**

Despite having been created on social media, research into the effects of online engagement on long Covid (LC) as a diagnosis is scarce. Studies on other health‐related communities and patient participation argue that social media and other digital technologies have been instrumental in creating new ways for activism, advocacy and sharing of experiences. With its status as patient‐made, LC constitutes an example of how diagnoses are (re)constructed through social interactions in addition to Western biomedical science and clinical practice. The aim of the study is to investigate the ways in which lived experiences and larger narratives of LC are communicated and form understandings of the condition as a heterogeneous diagnosis/phenomenon.

**Methods:**

This study uses netnography, mainly focused on hidden observations of patient support and advocacy forums in which users' posts are individually sourced and thematically analysed. These themes are further discussed to illustrate overarching discourse that contributes to the sense‐making and creation of knowledge surrounding LC.

**Results:**

The study highlights three major themes, namely: users seeking commonalities in experienced symptoms, interpatient tinkering as a form of biohacking conceptualised as an epistemological process, and negotiating expertise.

**Discussion:**

The study finds that narratives shared in online spaces regarding LC act as critical factors that serve not just the affected individuals' sense‐making and understanding of their lived experience, but also in the construction of the diagnosis itself. Through sharing experiences, symptoms, scientific information and treatment options, forum users contribute to knowledge production processes that change the definition of LC as a diagnosis. Building on the sociology of diagnosis, I argue that LC serves as a significant example of how diagnoses are products of the entanglement between biomedicine, clinical practice, the social and the digital.

**Patient or Public Contribution:**

The project operates under the premise that patients from different social, cultural and professional backgrounds participate in online discussions about long Covid and that these individuals work towards individual as well as collective well‐being. As such, highlighting their engagement has the potential to inform and strengthen research and clinical practice in various ways.

## 1 Introduction

In an article in Yale Medicine News, Dr. Lisa Sanders, Medical Director of Yale's Long Covid Multidisciplinary Care Center, says that: ‘We don't know enough about the cause of many of these symptoms to create a test for them. The problem is not with the patient with the symptoms, but of the science surrounding them […] If any good can be said to come out of this pandemic, it will be a better understanding of Long Covid and many of the other post‐acute infection syndromes that have existed as long as the infections themselves.’ [1]. Sanders' remarks highlight how a lack of scientific understanding of long Covid (LC) led to an inability to offer care and support to people affected by the condition. This, in turn, has forced many to find answers to their illness elsewhere, resulting in the conceptualisation of LC as a diagnosis [2] in online communities. As highlighted by Callard and Perego [2], the condition was coined on Twitter by Perego, herself, and was quickly adopted and shared by other patients. Eventually, it was acknowledged as a *formal* diagnosis in just a matter of months, though not without scepticism from some medical researchers regarding the *lay* origin of the name [2].

Despite its connection to digital spaces, research into the effects of online engagement on LC as a diagnosis is scarce. Studies on other health‐related communities investigate patient participation and argue that social media and other digital technologies have been instrumental in creating new ways for activism, advocacy and sharing of experiences. As such, LC constitutes an example of how diagnoses are (re)constructed in the intersections of experiential and Western biomedical expertise, as well as clinical practice.

The aim of this study is to investigate the ways in which experiences and narratives of LC are communicated in online forums and form understandings of the condition as a heterogeneous diagnosis/phenomenon. The focus lies on themes within online discussions and narratives connected to LC that shape the diagnosis. Several of the challenges that people suffering from LC are facing, such as inaccessibility to proper care, not being believed and dismissal, are not new and illustrate the continuation of contesting experiences of illness [3]. Individuals who express being met with scepticism and mistreatment attempt to find alternative ways to form an understanding of their illness. They put significant amounts of work into becoming an *e‐patient* [4] who possesses both experiential expertise and biomedical know‐how about their symptoms to break traditional clinical paradigms and piece together a diagnostic picture for themselves. However, this knowledge can only do so much for the individual in terms of treatment and must often be used to convince a medical professional that their illness is *clinically* diagnosable, lest the care seeker might not be deemed eligible for adequate treatment.

As argued by Claudia Egher, while using the internet as a technology to self‐monitor and exchange information regarding one's health can be welcomed, to what extent this should be encouraged to influence their care is debated [5]. Similar to online LC communities, Egher writes that inter‐patient discussions on mental health show that engaged individuals use these as means of support, as well as to construct treatment plans that are tailored to their specific needs [5]. Egher's work on mental health further connects to LC patients' efforts to manage symptoms. In lieu of clinically prescribed treatment, alternative plans are configured through processes of *tinkering*, as discussed by Natasja Kingod [6]. These configurations are results of *biohacking* and *do‐it‐yourself biology* covered in previous research [7] and are conceptualised in this article as collective *interpatient* tinkering: a trial‐and‐error‐based mode of knowledge production which, I argue, shapes the LC diagnosis itself.

Thus, a focal point for this article is on the theorisation about the construction of diagnoses, knowledge production and expertise, where the empirics are used to contextualise the potential that comes with considering social life on the internet as an important site of knowledge production. As such, this study attempts to answer calls from affected individuals and academic institutions alike [8], for research into patient communities that can highlight their frustrations and desperation connected to a lack of care and understanding from and within healthcare institutions.

## 2 Theoretical Framework

### 2.1 Sociology of Diagnosis

In clinical and laboratory work, diagnoses and diagnostic processes are an integral part of organising illness. As argued by Annemarie Jutel, diagnoses do ‘work’ in determining cause, potential outcomes and setting up treatment plans for individuals seeking care [9]. Jutel argues that this categorisation is done at the prerogative of health professionals, which often excludes experiential accounts for the benefit of objective and measurable science. This hierarchy of knowledge has, however, been subject to reconfiguration in recent years, in part due to the digitalisation of medical knowledge, which includes both lay and experiential expertise. Today, patients and practitioners have access to the same information bank(s) regarding disease and illness through personal computers and smartphones, which can be (re)interpreted and shared with others in an instant. Sarah Nettleton argues that this *informationalization* of medicine [10] means that practitioners' and patients' access to the same knowledge bank(s) informs their understandings of conditions and has the potential to (re)configure their compositions. A diagnosis, then, should not be seen as isolated within biomedical practice, but rather as a construct at the intersection of social relationships and different modes of knowledge [11]. For contested or unsettled diagnoses such as LC, the social aspects are particularly visible through interactions between individuals affected by the condition, as well as the advocacy and knowledge such interactions produce.

### 2.2 Digital Health Citizenship

To discuss the effects of the digital on understandings of LC, I connect sociology of diagnosis to Dimitra Petrakaki, Eva Hilberg and Justin Waring's concept *digital health citizenship* based on the idea that ‘digital technology produces health citizenship at the intersection of biosociality and technosociality’ [12]. The authors theorise that this novel type of citizenship emerges from the entanglement of digital technologies—health apps, online health services and so forth—with the concept of *patient activation*: the shifting of responsibility from governing bodies to the citizen for their own well‐being.

Connected to the idea of the bio‐digital, as posed by Alan Petersen, Allegra Clare Schermuly and Alison Anderson [13], digital health citizenship emphasises the unsettledness that comes from the development of an individual's view of their experienced condition in relation to technology. The concept of bio‐digital citizenship is key here as it expands on the social to include health work that occurs in digital spaces, which aims at building communities, attracting funding, promoting research [12], as well as using experiential knowledge to help others cope with their symptoms. LC communities show the potential of such processes and further how engagement with others, outside official healthcare institutions, shape and ‘unsettle’ the diagnosis. As LC is an extensively heterogeneous condition still under investigation from the biomedical perspective, it carries a great deal of uncertainty, which affects those with lasting and debilitating symptoms.

## 3 Materials and Methods

Empirics for this project are collected through netnographic enquiry, signifying the use of ethnographic methods in digital spaces. Here, the researcher assumes the role of an observer who follows the progression of an internet‐based forum and discussions within it for an extended period [14]. This study is further enhanced using a field journal. The empirics for the study were collected from three Reddit communities related to LC, which were chosen based on the number of members and frequency of activity. Data was collected from the time of ethics approval in May 2024 through November 2024. After familiarisation with the field and patterns begin to form, text posts and comments that are thematically connected are picked out manually and copied into a coding program for analysis. Selected posts have been coded, thematised and analysed following Braun and Clarke's six steps of *thematic analysis*, namely: Familiarisation with the discourse through the collected data, generating codes that describe different understandings of LC, creating overarching themes from the codes, reviewing themes to see if more/less are needed, defining and naming of themes, and communicating these through writing the text [15]. In total, the empirical data bank consists of approximately 200 individual posts, of which 14 have been used as examples in this article.

As the main method of data collection has been *hidden observations*, significant reflexive, ethical work has been put towards protecting the identities and integrities of those who share their stories. Following the netnographic work of Carolyn Wilson‐Nash, narratives collected from online forums have been rewritten and paraphrased to mitigate traceability to the original source [16]. This rewriting process entails restructuring sentences, changing verbiage and omitting information that could be used to identify a user, such as geographical locations. Further, all authors of posts have been given a pseudonym using a code structure, which specifies platform origin, date and a number for each designated user. Posts containing photos and names are disqualified from gathering, and original text posts with connected usernames are saved on a secure server service offered by Linköping University. The project has been approved before data collection by the *Ethical Review Authority* [2024‐02787‐01].

## 4 Results

### 4.1 Shaping Diagnoses Through Symptomatic Similarities

As has previously been pointed out, the symptomatic heterogeneity of LC is one of the aspects that has made the condition difficult to specify for clinicians, researchers and patients alike. A requirement for biomedical research is identifying specific biomarkers that set the condition apart from others, thus helping in solidifying the framework for what a clinical diagnosis might look like. Observing online engagement on LC shows that such processes are not limited to biomedical institutions.

To understand one's individual experiences of illness, users engage in sharing known and new symptoms in the hopes of finding common ground that can be connected to an LC diagnosis. Such posts illustrate the heterogeneity, complexity and unsettledness of the condition. Common symptoms, such as fatigue, shortness of breath, cognitive issues related to memory and concentration, and heart palpitations [17], appear to form a baseline for the illness among many users. However, since these symptoms are visible in other conditions as well, community members spend considerable time working through what additional aspects make their experience unique:

*R#50424:* I'm wondering if anyone else is struggling with sore or clicking noises from their joints? Maybe it's just because of not being able to exercise, but I've read that it could be a symptom in MCAS.

*R#11124:* I can't get my ears to stop hurting. Despite there being no infection or having any other illness that could relate to it, I feel like it could be related to my LC. Anyone else experienced this? Looking for advice.

Discussions to find common symptoms are found to be important in the co‐diagnostic process for two main reasons: First, it helps individuals find potential treatment options for themselves, and second, it works to affirm the presence of LC in one's body by differentiating it from other conditions, potentially opening for a clinical diagnosis.

LC's similarity to other contested conditions, such as myalgic encephalomyelitis/chronic fatigue syndrome (ME/CFS), post/chronic Lyme disease and fibromyalgia [18], is important to note here, as it is commonly brought up in conversation between forum participants. Conceptualising this process, the way LC is positioned against other conditions illustrates the importance of determining what LC *is* by establishing what it is *not*. In discussion on the similarities between LC and ME/CFS, for example, two commentors write:

*R#10524:* It's a bit of a leap to confidently say that people with ME/CFS and LC have the same underlying issues. The focus here should be on physiopathology and not on semantics.

*R#20524:* See the definitions here [WEBSITE]. If you don't meet the criteria for ME/CFS, you probably don't have it.

The similarities and differences between the conditions discussed here noticeably form a baseline for what can be expected from LC contra ME/CFS. Although contested in some public debates, the ME/CFS diagnosis holds a high status here because it is *settled* in the context of the LC forum. The symptomatic picture was decided and published through an official channel, rendering it less susceptible to reconfiguration than is LC. This theme is further discussed in a different conversation on the same topic, geared towards defining LC more specifically:

*R#30424:* You don't have ME/CFS if you don't have PEM. Simple as that.

*R#40424:* LC comes with multiple symptoms, and I think it's important that we stick together on this. I have both POTS and SFN so it's the ME/CFS variant.

The first commentor here sets a boundary between diagnoses using post‐exertion malaise (PEM) as a defining symptom of ME/CFS. The second commentor highlights that LC could be used as an umbrella term for *variants* that share symptoms with other conditions. Their mention of postural orthostatic tachycardia syndrome (POTS) and small fibre neuropathy (SFN) illustrates how their LC looks like ME/CFS, but they are still careful not to conflate the two. As reactions to a question of whether all post‐viral infection conditions could be one and the same, both posts illustrate the unsettledness of LC and, at the same time, validate ME/CFS.

As mentioned by Jutel and Nettleton, diagnostic processes involving lay expertise have historically been proven effective in relation to contested conditions, highlighting their potential to contribute to the establishment of new diagnoses [11]. Here, the patients' work to confirm and/or debunk specific aspects of the condition assists in narrowing the diagnostic framework. For instance, while the ME/CFS diagnosis is solidified in the above examples, knowledge about what fits the model of LC is produced through systematically *removing* it from other conditions. This type of description, comparison and reasoning taking place between community members has become indicative of the diagnostic process, as it might in a clinical or *traditional* laboratory environment. The Reddit forums, in this sense, become laboratories in themselves where not only symptoms inform understandings of diagnoses, but as will be shown, biomedical research papers and raw biological data, as well.

### 4.2 Interpatient Tinkering

The second theme of this article concerns the ongoing process of *interpatient tinkering*, where the users discuss potential treatment options for various symptoms, mostly focused on managing microbial health and blood values. Like Egher's article on bipolar patient communities, these ‘creative tactics’ [5] involve users offering recommendations based on experiences of biomedical, pharmaceutical, holistic and/or any combinations of treatments based on their own experiences. Reoccurring examples include antihistamines, low‐dose naltrexone therapy (LDN) [19], selective serotonin reuptake inhibitors (SSRIs) and over‐the‐counter dietary supplements such as *N*‐acetylcysteine (NAC): a compound primarily developed to be taken for paracetamol overdose, and here used to increase the presence of antioxidants in the body:

*R#20824:* I've tried pretty much everything that has been recommended to me, but nothing has had any real effect, but then I found this augmented NAC and it has helped me so much.

*R#10624:* I've noticed that a lot of people have given up on augmented NAC because it didn't do enough for them. IMO this might be because the standard dose isn't enough for everybody and that it is pretty spendy. […] I didn't get the effect I had hoped for with the standard dose either, so I incrementally increased it to about double which is when I started feeling a real change.

In Egher's meaning, these practices should not be seen as intentional attempts to ‘resist the current health regime*’* [5], but to do work based on a variety of health sciences to find ways of making their lives easier. This connects to a common critique from official health institutions about the potential pitfalls of patients relying on the internet for information. Granted, one does also find narratives that involve questioned, alternative treatment methods such as the infamous ingestion of Ivermectin: a medication that was popularised during the Covid‐19 pandemic for its suggested ability to cure infection from SARS‐CoV‐2. As an antiparasitic drug, Ivermectin has remained a controversial topic, which prompted the Federal Drug and Food Administration (FDA) to publicly remind the public that they were not, in fact, horses and should therefore not ingest the drug to treat Covid‐19. Despite the lack of *evidence* from the biomedical community, Ivermectin is one of several contested remedies that have found traction on the internet:

*R#10924:* …I decided to ask my doctor about prescribing Ivermectin and managed to get a few doses. I will say that it has reduced my LC, but it also came with its own side‐effects… Now my symptoms are back but I'm not sure I want to keep taking Ivermectin even though it helped a little. What should I do

Importantly, among the communities explored for this article, there is an overwhelming emphasis on *evidence‐based* biomedical knowledge, and the promotion of remedies such as Ivermectin is often met with immediate and stark scepticism. This again highlights the embracing, rather than rejection, of biomedical research, where tinkering could be viewed as relatively safe, which will be elaborated on further in the third theme.

Concerning all examples above, there is mention, either in the original post or comments, of specific treatments de facto being prescribed and/or recommended by a health professional. Further, in the absence of clinical assessment, often due to cost or lack of specialist care facilities, individuals are still able to acquire substances and tinker with doses and/or mixtures of other drugs, such as SSRIs, to manage their symptoms:

*R#31124:* Recently made the decision to start on [SSRI] after my regular antidepressants aren't cutting it anymore. Any pros and cons anyone has experienced with this?

*R#41124:* I've been taking the same thing for my [CONDITION] for years and the only downside was feeling bad initially … works great for symptoms other than depression. Like brain fog etc.

The discussion above hints that SSRIs are initially used to combat mental health issues, and later become part of tinkering with neurochemical processes for symptoms more closely related to LC. A common issue, brain fog, is here understood as a neurological imbalance which could, as illustrated by the users, reasonably be treated with substances meant to adjust other brain‐related conditions. A third commentor notes that there are other SSRIs that have been approved for treating LC in their location, validating the potential benefits of this experimental effort.

The culmination of this interpersonal tinkering is another piece in the ever‐changing puzzle of LC. Much like the negotiation regarding what LC is and is not in relation to other conditions, users will share advice, test and tinker with different options to find a remedy that makes their life closer to what it once was. In doing so, the community members also shape the framework for the molecular composition of the condition: if treatment A mitigates symptom 1 for enough people, then a potential biomarker could have been identified, as exemplified in this response to the example about clicking joints:

*R#60424:* Yeah, there's probably a connection to hypermobility of the neck here as I see more and more people with [DIAGNOSIS] popping up. Seems like too many to be a coincidence.

In contrast, if treatment A does not relieve symptom 1, then the chemical process tinkered with may not be a direct cause. This is, of course, an oversimplification of the epistemological processes at play, but works as an illustration for how tinkering positions itself ‘…on the crossroads of between biology, medicine, and culture…’ [7]. As made visible by discussions on treatment options, one finds the construction of knowledge of LC to be extensively experiential, yet also deeply entangled with expert biomedical knowledge, diffusing borders between modes of expertise in the making of the diagnosis.

### 4.3 Intersections of Expertise

What benefits LC from other contested conditions is its close entanglement with the SARS‐CoV‐2, which Barker et al. argue legitimises its position [18]. Covid‐19 undergirds LC because of a biomedically *evidenced* causality. Through the sharing of news articles and scientific journal articles, community members express the authoritative stance that Western biomedical science has within the discourse. The practice of using test results and sharing of biodata, which show deficiencies or surpluses in specific medically normalised levels of enzymes, proteins and/or irregularities on a molecular level, exemplifies this. Forum users use a combination of experiential expertise, health data and lay biomedical expertise that they have gathered throughout their illness to open an avenue for discussion. For example:

*R#11024:* Previous tests I've taken show increased levels in [ENZYME]. Some of the treatments I've tried to mitigate these have helped and didn't have an impact on perception. I would have [OTHER SYMPTOMS] but was able to successfully treat those with histamine agents. […] Lastly, I haven't had any issues with the stuff I've been eating but sugary drinks give me brain fog instantly.

This post is posed as a question on how to deal with memory problems and mental clarity, and answers often follow a similar narrative structure as above, namely: test result, tried treatment options and treatment results. These posts contain different nodes of expertise that come together and blur the boundaries between biomedical diagnostics and experiences of illness. The crowdsourced medical data presented by the users carries value to biomedical health research as it has the potential to ‘fill gaps in clinical practice that could not otherwise be identified’ [12], simultaneously validating the (lay) expertise of the community member. As such, they denote the production of health knowledge that Petrakaki et al. argues as foundational for digital health citizenship [12].

The level of expertise required to interpret the information shared is further evident by discussions on health data in the forum.

*R#20724:* I found out that DNA analysis can show mutations in the CCR5‐delta 32 gene which could indicate lower susceptibility to Covid. Anyone know more about this?

*R#21124:* I have noticed that my TSH values have gone from a standard range between [VALUE] down to hyper. Have been taking parts off my pills to mitigate.

Granted, due to the number of healthcare professionals affected by LC, it is likely that individuals with expertise in chemokine receptors and regular thyroid‐stimulating hormone levels frequent these communities, which would further add to the depth of the knowledge produced here. In either case, this detail in nomenclature and scientific lingo is commonplace, and users who may not be familiar with certain terms will simply ask for a definition. How the information is explained and interpreted is in the privy of actors involved and may or may not add to the configuration of their own illness.

Notably in the above example, the second commentor mentions that they have been adjusting the dosage of their regular medication to bring their TSH to its normal range and inquiring if others have had similar experiences. This constitutes an example of medicalisation that falls outside institutionalised healthcare, prompting a deepened familiarity with the molecularised self [20] and allows for pharmaceutical and/or holistic tinkering.

## 5 Conclusion

In this article, I have argued how contested diagnoses such as LC are intrinsically connected to the digital in an intersection of different modes of knowledge and expertise, such as biomedical, experiential and lay. Using the LC community members' own narratives about their experiences with symptoms, healthcare and different treatments and connecting them to Jutel's concept of the sociology of diagnoses, I have illustrated how the condition can be seen as a subject to various simultaneous diagnostic processes. The experiential has often been ignored to the detriment of health citizenship. In line with Petrakaki et al. [12], I argue that focusing more attention on the knowledge production that occurs in online spaces between people affected by the condition can not only offer insight into individual, and collective, experiences and understandings of LC, but also provide information on how patient work with comparing symptoms and interpatient tinkering can aid in the development of comprehensive treatment strategies and methods that expands the biomedical paradigm. LC is notoriously homogeneous in symptoms and hard to pin down, which has contributed to its unsettledness as a diagnosis, but seemingly becomes more stable through interpatient engagement. Regardless of prior expertise. Therefore, I would like to emphasise the potential of *patient‐inclusive* research projects, as the patients' experiential and lay expertise, as seen in digital spaces, offers unique perspectives on illness and symptom mitigation that need to be heard.

What can be seen in this study is that the trust in biomedical solutions and explanations still holds epistemic authority and that the making of different treatments is often discussed within such a framework. This is important to note that although the cause and treatment options for LC are still under investigation, many reported symptoms can still be treated through tried and tested clinical measures. As Sanders [1] points out in the article mentioned in Section 1, one of the first tasks for medical professionals is to reassure patients that there are parts of LC that do not show up in tests and that not being able to identify causality should not be a reason for dismissal. Here, further research into expertise in patient communities can enrich clinical knowledge and mitigate cases where patients and healthcare workers feel lost.

Additionally, in a study by Kingstone et al., informants express that they have sought information and explanations of symptoms through social media and that this has brought about further anxiety about their condition [21]. This highlights another significant factor in the understanding of LC, as it shows how online engagement can contribute to *worsened* symptoms that are not always visible in meetings with local healthcare institutions. Kingstone et al. draw the conclusion that a primary concern in the understanding and treatment of those seeking care is not always heard. It is thus pivotal that fieldwork is conducted in online spaces, acknowledging its role in knowledge construction, lest persons affected by LC and other debilitating post‐viral conditions disappear into the background.

These media platforms are spaces that people utilise when they express frustrations about a lack of information or misdiagnosis/mistreatment in their local healthcare system. In their struggle to understand and cope with their everyday life, they construct narratives and hypotheses aimed at helping themselves and others. As aptly put by Tuva Beyer Broch and Tom Bratrud, it is by embracing the digital turn in communication that we can form ‘…a better understanding of polarisation, identity formation, and the conditions for democracy at large’ [22]. Thus, I argue that studying the discourse and stories told in digital spaces regarding LC has the potential to inform understandings of experiences of illness, care and patient advocacy for the betterment of healthcare and policy work.

## Author Contributions

**Petter Almqvist‐Ingersoll:** writing – original draft, conceptualisation, investigation, methodology, validation, formal analysis, data curation.

## Ethics Statement

The project is approved by the Swedish Ethical Review Authority (Dnr 2024‐02787‐01).

## Conflicts of Interest

The author declares no conflicts of interest.

## Data Availability

Data supporting this study cannot be made available for ethical research reasons.
